# A gene mapping bottleneck in the translational route from zebrafish to human

**DOI:** 10.3389/fgene.2014.00470

**Published:** 2015-01-12

**Authors:** Niek de Klein, Mark Ibberson, Isaac Crespo, Sophie Rodius, Francisco Azuaje

**Affiliations:** ^1^NorLux Neuro-Oncology Laboratory, Department of Oncology, Luxembourg Institute of Health (formerly CRP-Santé), Luxembourg, Luxembourg; ^2^Vrije Universiteit Amsterdam, Amsterdam, Netherlands; ^3^Vital-IT Systems Biology Division, Swiss Institute of Bioinformatics (SIB), Lausanne, Switzerland

**Keywords:** zebrafish, zebrafish-to-human gene mapping, orthology inference, genome annotation, translational research

## Abstract

Among a diversity of animal models of disease, the zebrafish is a promising model organism for enabling novel translational biomedical research. To fully achieve the latter, a key requirement is to match molecular readouts measured in zebrafish with information relevant to health and disease in humans. A fundamental step in this direction is to accurately map gene sequences from zebrafish to humans. Despite significant progress in genome annotation, this remains an intricate and time-consuming challenge. Here we discuss major obstacles that we had to overcome to systematically map genes from zebrafish to human. We identified important disparities, as well as partial agreements, between five public zebrafish-to-human homology resources. There is still a need for standardized, comprehensive genomic mappings between zebrafish and humans. Without this, efforts to use zebrafish as a powerful translational research tool will be stalled.

## LAYING OUT THE ROAD

The zebrafish (*Danio rerio*) is a powerful and relatively low-cost tool for fundamental and translational biomedical research. It offers *in vivo* models with potential clinical relevance, which are valuable to elucidate disease mechanisms, novel therapeutic targets, and candidate therapeutics (Mione and Trede, 2010; Gemberling et al., 2013; White et al., 2013; Kikuchi, 2014). Substantial efforts to enable zebrafish research have been reflected in ongoing initiatives in the USA, Europe, and elsewhere^1^. Examples of recent significant outcomes include The Zebrafish Model Organism Database (ZFIN^2^; Bradford et al., 2011) and the publication of the zebrafish reference genome sequence (Howe et al., 2013). The latter estimated that about 70% of human genes have at least one unambiguous zebrafish ortholog (Howe et al., 2013).

Notwithstanding the quality and applicability of these advances, key challenges remain to translate findings from zebrafish to humans on the basis of genome-wide sequence mapping. In this translational route, we are facing a heavy bottleneck early.

## THE NEED FOR ACCURATE AND COMPREHENSIVE GENE MAPPING

A crucial task for investigating translational research applications is the linking of molecular readouts measured in zebrafish to information relevant to human health and disease. To accomplish this, a key requirement is the matching of gene sequences from zebrafish to humans at a genome-wide scale. This step goes beyond the automated conversion of gene symbols, and often involves the association between multiple homologous sequences that are included in gene expression microarrays or RNA sequencing experiments.

A realistic scenario begins, for example, with the identification of a set of genes that are differentially expressed between pathological and control states, e.g., disease vs. healthy phenotypes. The resulting gene list may be mapped to homologous genes in humans using HomoloGene^3^ (Wheeler et al., 2001; Acland et al., 2014) and ZFIN (Bradford et al., 2011). Additionally, researchers may require to map sequences from specific microarray platforms, for example: from Affymetrix’s GeneChip Zebrafish Genome Array to GeneChip Human Genome U133A (goo.gl/d3lCPL). This is an essential prerequisite to perform search and matching of “omics” profiles related to disease and drug responses, which are stored in different databases. Prominent examples of the latter are the Gene Expression Omnibus (GEO^4^; Barrett et al., 2013), ArrayExpress^5^ (Rustici et al., 2013) and the Connectivity Map (cMAP^6^; Lamb et al., 2006; Lamb, 2007).

## PRACTICAL CHALLENGES REMAIN

We recently implemented this process as part of a project in drug repositioning that applies the zebrafish as *in vivo* model of heart regeneration. In this particular case, we aimed to match sequence probes from zebrafish to human using the microarray chips indicated above. Initially, we expected that a single mapping resource, such as HomoloGene (Wheeler et al., 2001; Acland et al., 2014), could allow us to go from zebrafish gene symbols to human homolog symbols (e.g., Entrez database IDs) in a relatively straightforward way. After testing options available, we understood that a single resource does not provide up-to-date, comprehensive genome-scale mappings.

**FIGURE 1:**
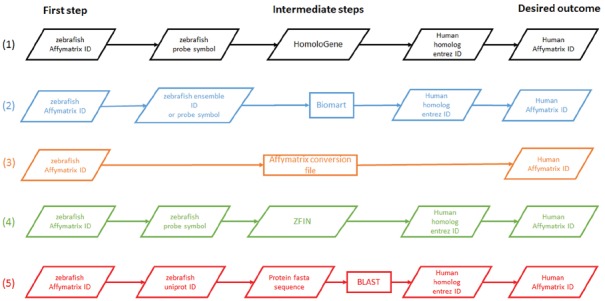
**Mapping zebrafish to human sequences via five annotated resources.** Sequence probes from Affymetrix’s GeneChip Zebrafish Genome Array were mapped to probes in GeneChip Human Genome U133A. Each mapping pipeline is based on a single resource independently: **(1)** HomoloGene (Wheeler et al., 2001; Acland et al., 2014), **(2)** Biomart (Kasprzyk, 2011), **(3)** conversion file provided by Affymetrix, **(4)** ZFIN (Bradford et al., 2011), and **(5)** BLAST homology searches performed at our laboratory (Altschul et al., 1990, 1997; Ye et al., 2006). In the latter method we focused on the most statistically significant BLAST match per query.

**FIGURE 2:**
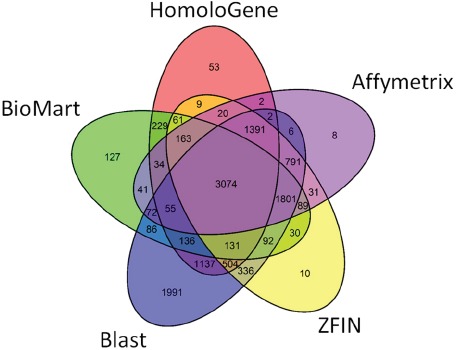
**Gene mapping agreement between the different homology annotation resources.** Number of mapped genes are shown for each resource and between-resource intersection.

To overcome this obstacle, we implemented a pipeline that incorporated five gene mapping resources: (1) HomoloGene (Wheeler et al., 2001; Acland et al., 2014), (2) Biomart^7^ (Kasprzyk, 2011), (3) conversion file provided by Affymetrix, (4) ZFIN (Bradford et al., 2011), and (5) BLAST homology searches performed at our laboratory (Altschul et al., 1990, 1997; Ye et al., 2006; Figure 1). Most of these resources (HomoloGene, ZFIN, and Biomart) apply a combination of: expert curation of orthology relationships found in the literature, manual orthology analysis, and (only) computational prediction of orthology. The other two resources are based on associations provided by the microarray manufacturer and our own computational predictions without deep expert curation. The human homolog Entrez IDs resulting from each procedure were compared, and overlaps among them were identified (Figure 2).

Using the five methods, a total of 12,593 human genes (Entrez IDs) were mapped from the 13,287 zebrafish sequence probes used as inputs (94.8%). Among the public resources, ZFIN (Bradford et al., 2011) reported the largest number of mapped genes (8533), followed by the Affymetrix conversion file (7580), HomoloGene (Wheeler et al., 2001; Acland et al., 2014) (7001), and Biomart (Kasprzyk, 2011) (6221). Our BLAST-based analysis (Altschul et al., 1990, 1997; Ye et al., 2006) generated 11,605 matches, including 1991 mappings that were not found in any of the other resources. Together, the five resources jointly agreed on 3074 mappings only (24.4%). Also there were relatively narrow overlaps between HomoloGene (Wheeler et al., 2001; Acland et al., 2014), a high-quality expert-annotated database, and the other resources (Figure 2). When two or more resources produced a mapping for any given zebrafish gene there was always an agreement in their mappings.

Considering these disparities and incompleteness, we decided to incorporate all available mapping evidence into the subsequent stages of our project. Although we assigned more confidence to mappings originating from expert-curated databases or to those supported by multiple resources (Figure 2), we also had to consider on a case-by-case basis those instances only mapped by a single source (e.g., Affymetrix file) or those only available in our BLAST search (Altschul et al., 1990, 1997; Ye et al., 2006). This semi-automatic, time-consuming conversion process was required for every zebrafish-derived candidate signature obtained in our project.

The major take-home message from Figure 2 is that whilst a large number of orthology predictions overlap between the five resources, this only amounts to just over 24% of the total number of annotated genes. This means that a straightforward voting approach only assigning orthologs commonly assigned by all resources, would mean ignoring 75% of genes, which is unacceptable for any genome-wide experiment. Even when taking just two resources such as Homologene (Wheeler et al., 2001; Acland et al., 2014) and ZFIN (Bradford et al., 2011), 1648 genes (24%) of the Homologene mappings are not found in ZFIN. The situation is even more striking considering ZFIN, where 3186 (37%) of mappings are not found in Homologene. Thus, the interpretation of whole genome experiments from zebrafish in a human context will be strongly affected simply by the choice of resource used for the orthology mapping.

## OVERCOMING OBSTACLES TO ENABLING RESEARCH

Our experience illustrates that performing zebrafish-to-human gene mapping remains a major challenge. This is a critical requirement for enabling research in different application domains. As zebrafish becomes a widely adopted genetic and systems biology model of disease, comprehensive and accurate zebrafish-to-human gene mapping represents a fundamental need. The complexity of this endeavor is magnified by intertwined evolutionary and genomics factors, including the considerable levels of gene similarity at the genome and gene family levels.

Finding maximal, high-quality sets of orthology relationships is currently constrained by the incompleteness of the zebrafish genome assembly, and in general by the evolutionary separation between species. Although our analysis considered (zebrafish) microarray probes that are linked to zebrafish genes previously annotated, this is an important factor to consider regardless of gene selection scheme or the lack of agreement between databases.

The *de novo* implementation of this process may be too time-consuming or even impractical for many laboratories, in particular those with limited bioinformatics resources. Moreover, even if bioinformatics capacity is available, the required information will continue maturing and evolving. This is very likely as the annotation of the zebrafish genome goes deeper and new evidence about gene function emerges. Furthermore, progress will be accompanied by a fast-growing interest in non-coding RNA sequences.

On one level, the agreement between gene homology resources highlight the confidence strength for such zebrafish-to-human mappings. On a gene-by-gene basis, databases that make major efforts to incorporate expert curation, ZFIN in particular, are likely to offer the highest quality relationships when those mappings are available. On another level, the considerable complementarity among these resources underlines the need for further annotation efforts, as well as their integration and standardization. Future comparisons could include other resources of orthology inference that were not considered in our analysis, such as the PANTHER classification system^8^. The incorporation of curation “evidence codes” (e.g., literature-extracted vs. manual orthology analysis) may also benefit the usage and integration of available resources. Future work could also benefit from incorporating phylogenetic evidence using multiple animal models and species.

Until a more standardized solution exists, researchers should not rely on a single resource for zebrafish ortholog mapping. Rather, we recommend using a combination of several resources and performing focused manual annotation on subsets of genes falling between annotation categories. In order to keep such numbers manageable this annotation could be restricted to small subsets of genes showing key biological relevance for the experiment in question. Ideally such annotation would be fed back into manually curated resources such as ZFIN (Bradford et al., 2011), thus making the annotations available to other researchers in the field. Researchers are welcome to request from us the multi-source mapping data discussed in this article.

The zebrafish can provide us with significant biological insights and novel directions for therapeutic interventions in a wide range of disease domains, including cardiovascular disease and cancers. To accomplish this vision, comprehensive and accurate zebrafish-to-human gene mapping is still necessary. Further public standardized efforts are needed. This will greatly depend on stronger support from research funders, researchers and other stakeholders.

### Conflict of Interest Statement

The authors declare that the research was conducted in the absence of any commercial or financial relationships that could be construed as a potential conflict of interest.

## Footnotes

1 https://zfin.org/zf_info/catch/zebrafish_programs.html

2 https://zfin.org

3 www.ncbi.nlm.nih.gov/homologene

4 www.ncbi.nlm.nih.gov/geo

5 www.ebi.ac.uk/arrayexpress

6 www.broadinstitute.org/cmap

7 www.biomart.org

8 www.pantherdb.org
